# Diet, Microbiome, and the Intestinal Epithelium: An Essential Triumvirate?

**DOI:** 10.1155/2013/425146

**Published:** 2013-03-17

**Authors:** Javier Rivera Guzman, Victoria Susan Conlin, Christian Jobin

**Affiliations:** ^1^Department of Pharmacology, University of North Carolina School of Medicine, CB 7032, 103 Mason Farm Road, Chapel Hill, NC 27599, USA; ^2^Center for Gastrointestinal Biology and Disease, University of North Carolina School of Medicine, CB 7032, 103 Mason Farm Road, Chapel Hill, NC 27599, USA; ^3^Department of Biology, Vertex Pharmaceuticals Inc., Laval, QC, Canada H7V 4A7; ^4^Department of Medicine, University of North Carolina, Chapel Hill, NC 27599, USA; ^5^Department of Microbiology and Immunology, University of North Carolina, Chapel Hill, NC 27599, USA

## Abstract

The intestinal epithelium represents a critical barrier protecting the host against diverse luminal noxious agents, as well as preventing the uncontrolled uptake of bacteria that could activate an immune response in a susceptible host. The epithelial monolayer that constitutes this barrier is regulated by a meshwork of proteins that orchestrate complex biological function such as permeability, transepithelial electrical resistance, and movement of various macromolecules. Because of its key role in maintaining host homeostasis, factors regulating barrier function have attracted sustained attention from the research community. This paper will address the role of bacteria, bacterial-derived metabolism, and the interplay of dietary factors in controlling intestinal barrier function.

## 1. Introduction

The gastrointestinal tract (GI) from the mouth to the rectum is lined by a single layer of cells that provides both physical protection from the potentially irritant and antigenic substances present in the luminal compartment and also performs essential biological functions such as absorption, secretion, and transport of various nutrients and water. In the lower GI tract, the intestine is divided into two distinctive anatomical sections: the small and large intestines. Importantly, the intestinal epithelium is constantly in a self-renewal state where proliferative stem-cell-containing crypts generate various specific cell lineages, namely, enterocytes, enteroendocrine cells, Paneth cells, and goblet cells. Biological events regulating intestinal epithelial cell proliferation, differentiation, migration, and survival are all implicated in the control of intestinal barrier function. Although, the distribution and ratio of these cells along the GI tract vary, collectively they protect the host against luminal contents; this single layer of cells forms a tight barrier preventing access of noxious substances to the underlining abundant immune cells. Moreover, the intestine is home to an estimated 10^14^ bacteria, termed the gut microbiota, which surpasses by a factor of 10 the estimated 10^13^ human cells. It is essential for host homeostasis to prevent an unregulated uptake/translocation of this microbiome, and the maintenance of an intact epithelial barrier plays a pivotal role in this function. There is significant interest in identifying factors and conditions influencing intestinal barrier function as these could have a profound impact on pathologies such as inflammatory bowel diseases (IBD) and colorectal cancer. 

The intestinal epithelium evolved in a unique environment where dietary metabolites, bacteria, and bacterial-derived metabolites are omnipresent. This environment likely provides a synergistic interaction between this tripartite that potentially influences each component. For example, the epithelium impacts microbial communities by producing various mucin products and antimicrobial factors that limit bacterial colonization and adherence. In addition, bacteria provide, as byproducts of their metabolism, various compounds (essential vitamins, antioxidants, short-chain fatty acid (SCFA), ect.) that impact host homeostasis [1, 2]. Finally, composition of dietary intake can also have significant impact on both the gut epithelial barrier and the bacterial communities [3–5]. 

 In this paper we focus on providing an overview of the latest emerging research that attempts to unify elements of these three fields: intestinal epithelial barrier, microbiome, and dietary intake—specifically, how these interact and modulate one another. We will discuss emerging studies into the molecular effects of short-chain fatty acids, their production by bacteria through intake of prebiotic fiber and resistant starches, and emerging details on probiotics and their mechanisms of action. 

## 2. The Intestinal Barrier

The mucosa surrounding the lumen forms a barrier to the microbiome and is comprised of a single layer of epithelial cells. An intact barrier is a prerequisite for normal health, and rapid resealing after injury is essential for prevention of disease [6, 7]. The epithelial barrier has the unenviable task of confining the microbiome and any potentially harmful substances to the lumen while regulating the flow of solutes, nutrients, and ions into the underlying mucosa [8, 9]. Transfer through an intact epithelium occurs by two routes: (1) across the apical plasma membrane via specialized channels (transcellular) and (2) through the paracellular space between epithelial cells via pores created by the paracellular junction proteins. The intercellular junctions consist of Zonula Occludens (tight junctions (TJs)) and Zonula Adherens (AJs) collectively known as the apical junction complex (AJC), gap junctions, and Desmosomes [10]. AJC formation confers cell polarity and selective barrier permeability. Maintaining barrier homeostasis requires the coordination of (1) the TJ proteins, (2) the actin cytoskeleton, (3) endocytosis, and (4) intracellular signaling pathways. In addition to these well-orchestrated processes, the commensal bacteria play an active role in maintaining host barrier homeostasis, likely by regulating cell renewal, promoting wound healing repair, and reorganizing the TJs.

Of all the transmembrane proteins (claudins, occludin, MarvelD3, JAM-A, tricellulin and lipolysis-stimulated lipoprotein receptor, LSR) [11–13], claudins determine the selective permeability of the barrier. This is achieved by different patterns of charged amino acids in the extracellular loops of individual claudin proteins, which interact to generate different sized pores through which solute transfer occurs [14–17].

While TJ stability is required for maintenance of barrier integrity, TJ formation has to be dynamic to accommodate intestinal epithelial cell turnover that occurs every 4-5 days [18]. To this end, TJ proteins are continuously internalized and recycled back to the plasma membrane via endocytosis. Under normal physiological conditions, the macroscopic renewal of TJs involves continuous strand breakage and reformation involving clathrin-mediated endocytosis [19, 20]. In contrast, claudins are recycled via a mechanism similar to that used for gap junction internalization, where TJ membranes are endocytosed together into one of the adjoining cells [21]. During internalization, the claudins separate from other TJ proteins and generate claudin-enriched vesicles, which have the potential to regulate the claudin composition of TJs.

TJ turnover and claudin expression can also be modulated by cytokines as a plausible mechanism for neutrophil migration across epithelial barriers [22]. In particular, TNF increased paracellular permeability *in vitro* by claudin downregulation [23]. Furthermore, cytokine-induced internalization of TJ proteins can be blocked *in vitro* using inhibitors of clathrin-mediated endocytosis [20]. TJ recycling can also be hijacked by pathogenic bacteria (e.g., *enteropathogenic E. coli, H. pylori*, and *C. difficile*) [24]. Bacterial-induced inflammation also increases claudin internalization and increases permeability [25, 26]. Macropinocytosis is another route in which TJ proteins can be internalized [27] and colocalize with markers of early and recycling endosomes. These data suggest a plausible mechanism for rapid redistribution of protein back to the TJ, sealing the epithelial barrier after an inflammatory insult has subsided [28].

Physiological regulation of barrier homeostasis relies on tightly controlled signal transduction pathways that converge on the cytoplasmic TJ proteins [29–36]. The cytoplasmic TJ proteins (ZO-1, -2, and -3; cingulin; and afadin) link the transmembrane proteins to the actin cytoskeleton and also act as scaffolds for major signaling complexes [29, 30, 37–39]. Phosphorylating components of the cytoskeleton, namely, myosin light chain (MLC) via myosin light chain kinase (MLCK) or Rho-associated kinase (ROCK), cause it to contract, which separates the TJ and increases paracellular permeability [28, 40–42]. In addition to the physical separation of the TJ, ROCK compromises barrier integrity by increasing endocytosis of TJ proteins [28]. Current opinion suggests regulation of TJs is a delicate balance between interacting networks incorporating protein kinase C (PKC), protein kinase A (PKA), mitogen-activated protein kinases (MAPK), and phosphoinositide 3-kinase (PI3-K) [42–45]. 

Though regulation of epithelial cell-cell junctions is an important factor for maintenance of homeostasis, a functional epithelium also requires regulation of IEC survival [46]. Differentiated cells traveling up from the crypt base (enterocytes, enteroendocrine cells, and goblet cells) to the villi are thought to die from anchorage-independent death (anoikis). However, recent findings show that at least a part of these sloughed-off cells can survive for a time after being evicted, giving credence to the hypothesis that these cells are sloughed off by simple lack of space due to cell overcrowding [47]. Additionally, apoptosis was believed to be the main regulator of intestinal epithelial cell numbers [48], given the strong *in vivo *staining patterns of caspase-3 in the gastrointestinal epithelium [49] and studies correlating caspase-3 and apoptosis in IECs shed from the intestinal monolayer [50, 51]. Mounting evidence supports, however, that the recently described necroptosis, or highly regulated programmed necrosis, is another active pathway that appears to regulate the intestinal epithelium homeostasis in response to different stimuli, including TNF-*α* which can also activate the apoptotic pathway [52–54]. Though born with seemingly normal epithelium, mice with the IEC-specific deletion of either caspase-8 or of Fas-associated protein with death domain (FADD), two proteins involved in cell death, quickly developed postnatal spontaneous phenotypes. IEC-specific deletion of caspase-8, for example, resulted in development of spontaneous ileitis with an 80% penetrance and was found to be responsible for TNF-*α*-induced necroptosis [52]. Mice with IEC-specific deletion of FADD showed reduced weight, diarrhea, and the development of spontaneous colitis, and the IECs of which were shown to have undergone necrotic cell death not apoptosis [55]. These findings indicate that numerous pathways regulate various aspects of IEC survival, a critical biological process for intestinal barrier function.

It is clear that alterations in normal signal transduction pathways that impact barrier homeostasis (proliferation/apoptosis/necroptosis) result in unregulated passage of luminal bacteria across the epithelium and subsequent aberrant activation of the mucosal immune system, leading to inflammation [40, 56, 57]. Increasing evidence also indicates that barrier function and its complex regulatory network are influenced by the microbiota and dietary components, both directly through endogenously produced microbial products, as well as indirectly through the metabolites in the host diet. 

## 3. Microbial Products and the Intestinal Epithelial Barrier

A wide array of pattern recognition receptors (PRR) are implicated in the sensing/detection of various microbial structures such as membrane components, nucleic acids, and motility apparatuses [58]. 

Toll-like receptors (TLRs) and Nod-like receptors (NLRs) are probably the most studied PRRs in the intestine, and their contribution to barrier function was investigated using various models of intestinal injury [59–61]. For example, TLR2 signaling through PKC is essential to enhance ZO-1-associated barrier function in intestinal epithelial cells following dextran-sulfate-sodium (DSS) exposure [62]. In addition, TLR4 and the signaling protein MyD88 have been shown to play a beneficial role in wound healing responses and restoration of barrier integrity in DSS-induced acute injury [63]. In addition, deletion of signaling molecules downstream of TLRs such as nuclear factor kappa B (NF-*κ*B) essential modulator (NEMO), the NF-*κ*B transcriptional subunit RelA, TGF-*β*-activated kinase, and other I*κ*B kinases within the intestinal epithelium results in increased susceptibility to colitis [64–67].

Although these findings highlight the important role of microbial structures in regulating barrier function, another layer of complexity is the relationship between the bioactive potential of the microbiota and the intestinal barrier. Indeed, the identification of specific microorganisms producing compounds involved in the modulation of intestinal barrier function has gained tremendous attention. 

Microorganisms and their associated genome (~3 × 10^6^ genes) are likely to produce compounds that shape host response. Indeed, the beneficial effects of lactic-acid producing organisms in fermented milk products on health were first proposed at the beginning of the 20th century by Metchnikoff [68]. Fermented milk products (FM) are representative of a group of natural compounds and microorganisms known as probiotics, which are defined as food supplements that are intended to improve health [69]. Probiotics have gained enormous interest in recent years as a means to help maintain intestinal homeostasis and/or alleviate specific GI pathologies [70–75]. In fact some strains of probiotic microbes can reduce gut permeability through direct effects on intestinal epithelial cells and reduce inflammation [5, 76–78]. Probiotics can mediate their beneficial activity through several mechanisms including (a) competitive exclusion of bacterial adherence and/or translocation; (b) release of bacteriocidin and lactic acid, which can inhibit the growth of pathogens; (c) production of butyric acid; (d) antioxidative effects; (e) enhancement of barrier function; (f) modulation of immune cell response; and (g) inhibition of NF-*κ*B activation [79–87]. We will discuss several of the most prominently emerging probiotic foods and microorganisms that impact the intestinal barrier.

Although probiotics have a relatively safe track record in humans, some studies have raised concerns about introducing billions of bacteria into a host [88, 89]. In an effort to circumvent this potential health hazard, attention has been directed on identifying probiotic-derived beneficial molecules that can be used in lieu of whole, live microorganisms. A recent report has shown that a recombinant 40 kDa soluble protein derived from *Lactobacillus rhamnosus GG *(LGG) is able to reproduce the antiapoptotic effect of the bacterium *in vitro*, a process mediated through an EGFR-dependent mechanism [73]. Importantly, the delivery of LGGp40 to the colon *in vivo* using a novel pectin/zein hydrogel bead system, is able to ameliorate DSS-induced intestinal injury as well as oxazolone-induced Th2-driven colitis [73]. Administration of supernatant from LGG cultures (LGG_sup⁡_), prior to oral gavage with ethanol, significantly ameliorated the multiple alcohol-induced damaging effects to the ileal epithelium. The protective effect of LGG_sup⁡_ on ethanol-induced increased barrier permeability was multi-factorial. It reversed the ethanol-mediated downregulation of TJ proteins ZO-1, claudin, and occluding, among others, and the mucosal protective proteins ITF, CRAMP, and P-gp mRNAs. In addition, LGG_sup⁡_ reversed the alcohol-induced decrease of Hif-2*α* mRNA and protein levels. As the mucosal protective proteins ITF, CRAMP, and P-gp are under Hif transactivational control, this suggests that maintenance of this transcription factor may play a strong role in LGG_sup⁡_-mediated effects.

Another lactobacillus with therapeutic potential is *L. brevis SBC8803*, which, unlike LGG, reportedly has beneficial effects when administered as a heat-killed, freeze-dried purification of monocultures. In a recent report, heat-killed *L. brevis *was able to dose-dependently induce heat shock proteins (Hsp) 25, 27, and 70 *in vitro* using the colonic cell line, Caco-2, Hsps, being important stress-induced proteins involved in the protection of the colonic epithelium against bacterial-induced injury [90–92]. Daily transanal administration of 0.1% freeze-dried *L. brevis* culture in saline decreased DSS-induced intestinal inflammation, improved survival, and decreased expression of TNF-*α*, IL-12, and IL-1*β* [72], highlighting the importance of identifying the differing mechanisms of action of different Lactobacilli. 

*Lactobacillus plantarum *(*Lp*) is a probiotic that has been the subject of numerous studies on the human GI system since the early 1990s [93]. This bacterium, which has been isolated from both healthy human intestine and the more potent strain isolated from sourdough (isolate 299V, aka DSM 9843), appears to have many beneficial effects in both animal models [94, 95] and human studies [96, 97]. Recent studies have further supported the potential of *Lp *in treating intestinal disorders. A small, 40-patient randomized, double-blind clinical trial showed that *Lp *resolved abdominal pain in all IBS patients compared to the control group and provided significant bowel movement regularity to constipated patients [96]. A recent, large scale follow-up clinical trial was performed using similar parameters and metrics and demonstrated beneficial effects [98]. While these and other studies focused on the ability of *Lp *to ameliorate overall disease either in animal models or in clinical trials, the underlying molecular pathways have just recently begun to be elucidated. Using a rat model of obstructive jaundice, analysis of the terminal ileum has provided a fairly thorough molecular characterization of the effects of a twice-daily oral gavage using *Lp*. Specifically, the authors found that *Lp* lowered obstructive jaundice-mediated intestinal epithelial cell (IEC) apoptosis and importantly increased mRNA expression of TJ proteins claudin-1 and -4, occludin, and ZO-1, in addition to PKC. Markedly increased JAM-A and PKC protein levels were also reported [99]. This increase in TJ proteins is consistent with an earlier study, which revealed intake of *Lp *maintains the intestinal barrier integrity in rats exposed to *E. coli *by inhibiting the *E. coli*-induced increase in barrier permeability [94]. Given the above-mentioned studies, one can assume this latter effect is likely through the increase of TJ proteins. Notably, *Lp *has also been suggested to release factors that significantly inhibit pathogenic bacterial adhesion to the mucosa [100]. Hence, the beneficial effect of *Lp* appears to center on upregulation of TJ proteins to strengthen the barrier, though it may also work to keep pathogens from adhering to the epithelium and invading the host. In addition, the beneficial effects of bacteria could be mediated through production of metabolites such as SCFA generated from the host diet. 

## 4. Bacteria-Produced SCFA and the Intestinal Epithelium Barrier

The anaerobic environment of the intestine allows certain gut microbes to harness nutrients through fermentation of nutrients passing through the lumen, resulting in the generation of a large array of metabolites. Among the metabolites produced by this process are essential vitamins such a vitamin K and most of the water-soluble B vitamins such as biotin, cobalamin, and riboflavin [101], which are then absorbed by the host [102]. Also among these metabolites are the SCFA, such as propionate, acetate, and butyrate derived from dietary fiber, fermentable carbohydrates, and resistant starches, which are not broken down in the upper digestive tract [103]. Fermentation of dietary fiber is important to intestinal homeostasis as this process induces upper gastrointestinal motility [104] and the satiety hormones glucagon-like peptide-1 (GLP-1) and peptide YY (PYY) [105–108]. In addition, not only do these SCFAs show therapeutic potential in treatment of patients with IBD [109, 110], but these bacterial derivatives also appear to further improve colonic health [111]. Interestingly, patients with ulcerative colitis (UC) appear to have impaired butyrate metabolism [112]. As such, the molecular mechanisms of action of these SCFA have become a subject of increasing investigation.

 The presence of SCFA in the intestine directly affects barrier permeability. Butyrate, for example, has been shown to protect Caco-2 cell monolayers from *Campylobacter jejuni* invasion and translocation in a concentration-dependent manner by increasing transepithelial electrical resistance (TEER) [113]. Similarly, butyrate, but not a mix of butyrate, acetate, and propionate, was shown to significantly reverse the increases in intestinal permeability, bacterial translocation, and histological damage caused by exposure to the chemotherapeutic agent 5-Fluorouracil in mice [114]. Unlike the first study, by using T-84 and Caco-2 monolayers, TEER was shown to be increased by all three individual SCFAs or by a mix thereof [115]. The ability of butyrate to increase TEER may relate to its capacity to increase cingulin, ZO-1, and ZO-2 proteins and mRNA levels as shown in Rat-1 fibroblasts [116]. In the same study, butyrate was shown to increase protein levels of cingulin in COS-7 cells and both cingulin and occludin in HeLa cells [116]. These findings suggest that SCFA strengthen the barrier through increase of both TEER and TJ protein production.

 The intracellular signaling events induced in IECs by SCFA, presumably by binding to their cognitive G-protein coupled receptors 41 and 43 (GPR41 and GPR43, resp.) [117] and their role in barrier function, remain elusive [118, 119]. For example, the protective effects of butyrate against chemical-induced damage and microbial translocation were recently shown to be associated with decreased I*κ*B phosphorylation (and presumably NF-*κ*B activity) [120], with the latter having been shown to play both positive and negative roles in maintenance of intestinal homeostasis [121]. Specifically, using a T84 human colon cell model of barrier function, butyrate was shown to protect against dinitrophenol-induced mitochondrial damage and increased permeability, as well as *E. coli *translocation. A more recent report has shown that butyrate activates the cyclic adenosine monophosphate (cAMP) → protein kinase A (PKA) → cAMP responsive element binding protein (CREB) pathway in Caco-2 cells [122]. However, butyrate had no effect on adenylyl cyclase or phosphodiesterase, enzymes that regulate production and degradation of cAMP, respectively. This observation is important because these enzymes are regulated by GPR signaling [123], suggesting that activation of the cAMP pathway by butyrate is independent of GPR41/43 signaling. As such the roles of GPR41 and GPR43 in SCFA signaling remain in question, though we are slowly gaining a better understanding of the mechanisms mediated by these formerly orphan receptors. 

Besides investigation of the molecular pathways involved in SCFA signaling, identification of bacteria and groups of bacteria producing SCFA, as well as the dietary component influencing them, has gained attention. 

The characterization of the complex microbial ecological system present in the intestine using ribosomal 16S sequencing techniques has revealed that the human microbiome is dominated by two phyla, the Firmicutes (~75%) and Bacteroidetes (~20%), with lesser contributions from Proteobacteria and Actinobacteria [124–126]. The Firmicutes found in the mucosal tissues are primarily composed of *Clostridium* XIVa and IV groups, which are active producers of SCFA. It is interesting to note that levels of acetic, butyric, and propionic acids decreased in fecal samples of IBD patients compared to normal healthy controls [127, 128]. Similarly, a number of reports showed that *Clostridium* XIVa and IV groups decreased in patients with Crohn's disease [129, 130]. These observations suggest that environmental conditions (diet, inflammation, ect.) could shape microbial status and influence their ability to produce SCFA. In addition, different pH levels are found throughout the colon and the fermentation of dietary fiber that produces SCFA is thought to be responsible for the low pH found in the proximal colon [131]. A recent study found that the majority of *Bacteroides* and *Proteobacteria *species were growth-inhibited at a pH of 5.5, a level representative of the proximal colon pH. In contrast, the majority of Gram-positive, both *Actinomycetes *and* Firmicutes* species, were tolerant to the lower pH. The latter is important as the *Firmicutes *clusters studied included butyrate-producing bacteria of the class *Clostridia *such as *Eubacterium rectale *and *Roseburia inulinivorans*. Moreover, using human faecal samples, it was shown that keeping fermentation chambers at different pH resulted in strikingly different bacterial profiles. While at pH 5.5 the majority of bacteria detected were *Firmicutes*, mainly *Clostridia *species, at a pH of 6.5 the majority of bacteria detected were *Bacteroides* species. Therefore, pH levels may shape the gut microbial communities by allowing growth of low pH-tolerant bacteria such as butyrate-producing *Firmicutes *species [132]. 

The ability of diet to modify the gut microbiota and SCFA production was also recently studied using obese patients given three diets comprised of successively lower carbohydrate levels, a source for bacterial SCFA production. These diets were administered for four weeks successively, and at the end of each 4-week feeding period stool was collected. The study showed that by lowering carbohydrate levels, also representative of high protein/low carbohydrate weight loss diets, total SCFA production was significantly and concomitantly reduced, with a notably disproportionate reduction in butyrate levels. And while the gut microbiota was not significantly altered in terms of *Firmicutes *versus *Bacteroides* ratio, a significant reduction in butyrate-producing *Roseburia *species and *Eubacterium rectale *was found to correlate with decreasing carbohydrate consumption [133]. The gut microbiome of children in Europe (EU) who consume mostly Western diets and that of children in rural Africa, specifically Burkina Faso (BF), whose diet is rich in fiber, were recently compared. BF children not only had higher levels of *Firmicutes* species but also of *Bacteroides* species in the genera *Prevotella* and *Xylanibacter*, with the latter producing enzymes that can hydrolyze cellulose and xylan, which the human enzymatic repertoire lacks. These latter species were completely absent in the EU children, and as predicted children in BF had higher SCFA levels than their European counterparts [134]. 

Fermentation itself, which results in SCFA production, has become a point of interest and recently two groups have studied the metabolites formed from the fermentation of human fecal samples in continuous 3 vessel spill-over systems which simulate the proximal, transverse, and distal colons of human [135]. The first group utilized a large amalgamation of fibers as the source for metabolite production and was able to show that when more fiber was introduced into the system at 3-fold that of baseline, an increase in colonic fermentation and concomitant increase in saccharolytic bacteria were observed in the fecal samples [136]. The second group took a different approach, however, opting instead to utilize human fecal samples and exposure to four different naturally high fiber-containing flours. While all flours had significant effects on resulting changes to the metabolite profile, each resulted in distinct responses from the samples. The “Pulses” flour (50 : 50 lentils and chickpeas) had the most promiscuous effect on the metabolites measured, showing increases in acetate, propionate, and tyrosine levels but decreases in butyrate, isovalerate, and trimethylamine levels. In contrast, the least effective flour, whole grain rye, only produced a significant decrease in the metabolite methanol, demonstrating how differently distinct fiber sources can act on shaping the SCFA and metabolite profiles [137]. Further studies will be needed to identify what fiber sources are optimal for SCFA production and associated beneficial effects. 

## 5. Milk, Bacterium, and the Gut Epithelial Barrier

 While identification of bacteria that provide benefit to the gut has been the subject of intense research focus, the role the diet has on promoting or inhibiting growth of detrimental or pathogenic bacteria has also become a burgeoning field. Although consumption of fermented milk has historically been associated with beneficial effects [68], as discussed above, it is clear that milk itself can have different effects depending on the source from which it is derived. For example, unlike human milk, animal milk, which forms part of the typical Western diet, does not contain the antimicrobial enzymes lactoferrin and lysozyme, which are thought to help shape the composition of the gut microbiota [138, 139]. This proof-of-principle was shown in a recent study using goat milk containing human lysozyme (HLZ), produced via a transgenic goat model, as HLZ was able to cause a compositional change in the gut microbiota of pigs. Specifically, after 17 days of being fed HLZ these young pigs contained significantly lower populations of *Firmicutes *and *Clostridia *species as compared to controls. In addition, though not statistically significant, the authors observed an increase in the *Proteobacteria* population [140]. This last finding is very interesting when taking into account a more recent study showing that consumption of animal-derived milk fat is able to alter the composition of gut microbiota communities in wild-type and *Il*10^−/−^ mice, a genetically susceptible model of colitis. Milk fat was able to induce a bloom in the population of *Deltaproteobacteria *within the *Il*10^−/−^ mice, specifically they were able to show a bloom of *Bilophila wadsworthia*, which coincided with an increase in taurine-conjugated bile acids in these mice. As bile acids are a source of energy for *B. wadsworthia*, it was then shown that gavage using bile acids in lieu of milk induced the same *Deltaproteobacteria *bloom. A bloom *of B. wadsworthia* by use of either agent also caused a significant increase in the incidence of colitis within the *Il*10^−/−^ mice [1]. Interestingly, increase in Proteobacteria is associated with IBD in humans [141, 142]. Further studies would be necessary before establishing cause/effect relationship between milk fat consumption, Proteobacteria, and IBD. 

In contrast to the detrimental effects that milk fat has in mice genetically susceptible to colitis, it has also been shown that *fermented *milk in turn can have beneficial effects on colitis models. For example, using a *T*-*bet* ^−/−^/*Rag*2^−/−^ model of UC, it was recently shown that administration of *B. animalis *subspecies *lactis*-containing fermented milk product (FM) was able to significantly reduce intestinal inflammation [143]. Amelioration of colitis was characterized by an increase in the population of lactate-consuming butyrate-producing bacteria species, as well as an increased presence of other SCFAs. Furthermore, the increase in lactate-consuming bacterial species correlated with a significantly reduced cecal pH. While this may seem inconsequential, low pH has been shown to create an inhospitable environment for *Enterobacteriaceae *species to grow, with the latter having been recently reported to be colitogenic in *T*-*bet* ^−/−^/*Rag*2^−/−^ mice [144]. Further support for the beneficial effects of *B. animalis *subsp. *lactis* was shown in another recent study that used a rat model of stress and hypersensitivity. FM containing *B. lactis* was able to reduce visceral hypersensitivity and stress-induced blood endotoxin levels; additionally FM was able to reverse stress-induced downregulation of TJ proteins JAM-A and occludin. Though interesting, the contribution of *B. lactis* to this effect is questionable given that the authors used FM containing not only *B. lactis *but also *Lactococcus lactis *CNCM I-1631, *Lactobacillus bulgaricus,* and *Streptococcus thermophiles *[71].

These studies represent an interesting point, showing how a seemingly innocuous food product such as milk can have far reaching consequences on an individual's gut microbiota. While the direct effects on barrier function were not investigated in these studies, the use of colitis models provides at least a reference point for potential beneficial effects and will help define molecular mechanisms of action.

**Figure 1:**
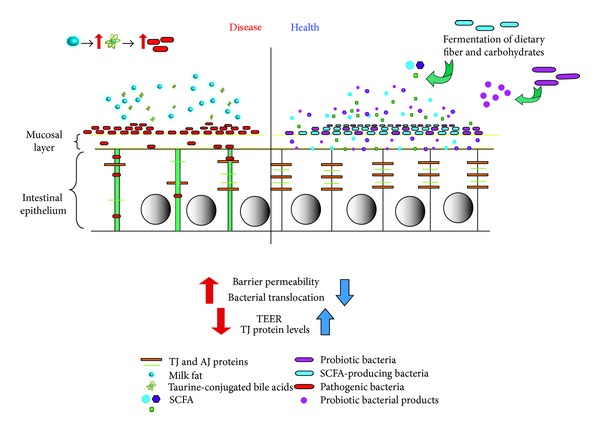
Intestinal epithelial responses to diet and microbes. Diets containing fermentable fibers, resistant starches and the like result in increased gut fermentation and SCFA production. A constant diet containing these elements would shift the host gut microbiome to increase the proportion of SCFA-producing bacteria. In turn, increase in SCFA production would also increase protection of the epithelium through strengthening the barrier as mediated by increased TJ protein production and TEER, as well as decreased permeability and bacterial translocation. Similarly, a diet containing probiotic bacteria would in time increase barrier function and integrity. Conversely, diet that promotes the increase in populations of pathogenic or opportunistic bacteria (as with intake of milk fat) within the landscape would have the opposite effects, decreasing TJ protein production and altering their distribution, as well as decreasing TEER and thereby compromising barrier integrity. This would then result in increased barrier permeability resulting in increased bacterial translocation and thereby increasing pathology such as increased intestinal inflammation.

Though the gut barrier landscape is a very complex environment, taken together, we can now envision it as one in which intake of diet affects the gut in one of two ways. On the one hand, it can affect the gut by promoting the increase of pathogenic or opportunistic bacteria and thereby damaging the barrier through increases in permeability and bacterial translocation, along with decreases in TJ proteins and TEER resulting in pathology such as inflammation. On the other hand, a diet that includes probiotic bacterial species or prebiotic fibers that result in SCFA would strengthen the epithelial barrier by increasing TJ proteins and TEER, as well as decreasing permeability and bacterial translocation helping to avoid or ameliorate pathology (Figure 1). 

## 6. Perspective

 The impact of bacteria on intestinal barrier function is clearly illustrated by the action of specific pathogenic enteric bacteria that have evolved remarkable means to penetrate and circumvent this important host defense mechanism. Pathogenic enteric bacteria such as *Salmonella*, *Shigella,* and *Yersinia *species, utilized specific effector proteins to alter intestinal tight junction proteins and weaken barrier function. On the other hand, millions of years of evolution have led to the acquisition of a complex intestinal microbiota that was selected for its capacity to maintain a symbiotic relationship with the host. This biota has formed through a complex set of environmental factors including dietary habits. Evidence suggests that this biota not only prevents pathogenic bacteria from accessing the epithelial barrier, but also actively promotes the state of a healthy barrier through the action of their metabolism.

 Some bacteria such as *Lactobacillus plantarum *appear to modulate the epithelial barrier through the action of secreted protein (*LGG *p40) whereas other such as Clostridium likely influence the barrier through production of metabolites (SCFA). In view of the richness and diversity of the microbiota, it would be important to “mine” this biota and identify microorganisms with “barrier protective function.” Because of the interplay between diet and microbial composition, identification of nutritional components that contribute to barrier function should also be a forefront priority. Integration of microbial genomic, metabolomics, and transcriptomic technology would be essential to carry this mission forward. Understanding the intricate relationship between epithelial barrier, microbe, and diet would undeniably contribute key knowledge that could be harness for therapeutic purpose.
